# Analysis of the Genetic Relationship between Atherosclerosis and Non-Alcoholic Fatty Liver Disease through Biological Interaction Networks

**DOI:** 10.3390/ijms24044124

**Published:** 2023-02-18

**Authors:** Francisco Andújar-Vera, María Ferrer-Millán, Cristina García-Fontana, Beatriz García-Fontana, Sheila González-Salvatierra, Raquel Sanabria-de la Torre, Luis Martínez-Heredia, Blanca Riquelme-Gallego, Manuel Muñoz-Torres

**Affiliations:** 1 Instituto de Investigación Biosanitaria de Granada (ibs. GRANADA), 18014 Granada, Spain; 2 CIBER on Frailty and Healthy Aging (CIBERFES), Instituto de Salud Carlos III, 28029 Madrid, Spain; 3 Department of Computer Science and Artificial Intelligence, University of Granada, 18071 Granada, Spain; 4 Andalusian Research Institute in Data Science and Computational Intelligence (DaSCI Institute), 18014 Granada, Spain; 5 Endocrinology and Nutrition Unit, University Hospital Clínico San Cecilio, 18016 Granada, Spain; 6 Department of Cell Biology, University of Granada, 18016 Granada, Spain; 7 Department of Medicine, University of Granada, 18016 Granada, Spain; 8 Department of Biochemistry, Molecular Biology III and Immunology, University of Granada, 18071 Granada, Spain; 9 Department of Preventive Medicine and Public Health, University of Granada, 18016 Granada, Spain

**Keywords:** atherosclerosis, non-alcoholic fatty liver disease, protein–protein interaction network

## Abstract

Non-alcoholic fatty liver disease (NAFLD) seems to have some molecular links with atherosclerosis (ATH); however, the molecular pathways which connect both pathologies remain unexplored to date. The identification of common factors is of great interest to explore some therapeutic strategies to improve the outcomes for those affected patients. Differentially expressed genes (DEGs) for NAFLD and ATH were extracted from the GSE89632 and GSE100927 datasets, and common up- and downregulated DEGs were identified. Subsequently, a protein–protein interaction (PPI) network based on the common DEGs was performed. Functional modules were identified, and the hub genes were extracted. Then, a Gene Ontology (GO) and pathway analysis of common DEGs was performed. DEGs analysis in NAFLD and ATH showed 21 genes that were regulated similarly in both pathologies. The common DEGs with high centrality scores were ADAMTS1 and CEBPA which appeared to be down- and up-regulated in both disorders, respectively. For the analysis of functional modules, two modules were identified. The first one was oriented to post-translational protein modification, where ADAMTS1 and ADAMTS4 were identified, and the second one mainly related to the immune response, where CSF3 was identified. These factors could be key proteins with an important role in the NAFLD/ATH axis.

## 1. Introduction

Non-alcoholic fatty liver disease (NAFLD) is a disease characterized by the excessive accumulation of lipids (steatosis) in the liver unrelated to any viral infection or excessive alcohol consumption [1,2,3]. NAFLD currently encompasses several liver conditions of varying severity, ranging from simple steatosis or steatohepatitis to the development of cirrhosis or hepatocellular carcinoma [4,5,6], and it is one of the most common liver diseases in the West, with a prevalence of 20–30%, rising to 70% in people with obesity or diabetes [1,4]. During the last decade, NAFLD has gained importance as a hepatic manifestation of the metabolic syndrome, for which it was renamed in 2020 as Metabolic Dysfunction-Associated Fatty Liver Disease (MAFLD). MAFLD differs from NAFLD in that it is identified on the basis of positive diagnostic criteria. MAFLD is diagnosed when patients present steatosis ≥ 5% in addition to at least one of the following: type 2 diabetes, obesity/overweight, or metabolic risk abnormalities (evidences of metabolic dysregulation) [7]. Moreover, MAFLD share similar pathogenetic molecular mechanisms that cause vascular damage, so it has been related to cardiovascular disease (CVD) and a cardiovascular risk (CVR) [8,9,10,11,12].

On the other hand, atherosclerosis (ATH) is a chronic, progressive disease affecting medium and large arteries. This inflammatory vascular disease has a lipid origin and is characterized by the thickening and hardening of the arterial walls due to the development of lesions and the accumulation of lipids and fibrous elements in them, forming atherogenic plaques [13,14,15]. The rupture of these atherogenic plaques is likely to cause thrombosis and major cardiovascular complications, such as myocardial infarction and strokes, which are among the leading causes of death worldwide according to the World Health Organization (WHO) [16]. It has been known for some time that there is a certain relationship between NAFLD and ATH, as both of them form part of the factors that make up the metabolic syndrome. Although it has not been possible to demonstrate a causal relationship, it is known that NAFLD promotes the onset and development of ATH in different ways [4,17], mainly by systemic metabolic aberrations, such as dyslipidemia, oxidative stress and inflammation, and liver-related elements, such as synthesis of hepatokines and coagulation factors [17,18].

The increased production of reactive oxygen and nitrogen species (ROS and RNS) resulting from mitochondrial dysfunction in the liver leads to increased lipid peroxidation, and in 20% to 80% of cases, the development of atherogenic dyslipidemia [1,19]. This leads to increased synthesis of LDL lipoproteins, chylomicrons, and very low density lipoproteins (VLDL), which are secreted by hepatocytes into the bloodstream and transformed into smaller, denser particles, sdLDL, which promote the formation of atherogenic plaques [1,17,19,20,21,22,23].

In arteries, the increased ROS level associated with NAFLD damages the endothelial cells, whose membrane phospholipids become peroxidized, promoting increased permeability in the tunica media of blood vessels and an increase in both inflammatory cell infiltration and vascular smooth muscle cell (VSMCs) proliferation and differentiation. This process appears to be related to NAFLD-induced angiotensin II overexpression [17,24] and other coagulation factors such as FII, FX, and FXII [17,25,26,27]. In addition, the proinflammatory factors IL-1β and TNF-α induced by ox-LDL promote changes in the components of the extracellular matrix surrounding the VSMCs contributing to the dedifferentiation of these cells [17,28,29]. Finally, the level of bioavailable nitric oxide, which exerts a protective effect on blood vessels [17,30,31,32], is reduced due to the action of ox-LDL in NAFLD [33,34]. This results in endothelial dysfunction preceding the development of atherosclerotic lesions [16,17,35,36,37,38,39,40].

Although recent studies and meta-analyses have described some of the molecular and cellular mechanisms that may explain the link between NAFLD and ATH, much remains to be explored in this field. In this context and considering the great socioeconomic impact of these two pathologies worldwide, bioinformatics studies could help to identify and inform us about the common molecular pathways involved in the development of ATH and NAFLD with early therapeutic and preventive purposes. In this study, a bioinformatic analysis of interaction networks has been carried out in order to identify the common factors between both pathologies contributing to the characterization of potential molecular pathways involved in the NAFLD/ATH axis.

## 2. Results

### 2.1. Data Acquisition and Visualization and Identification of Differentially Expressed Genes

The gene expression profiles by array GSE89632 and GSE100927 were selected to identify the differentially expressed genes (DEGs) for NAFLD and ATH, respectively.

The GSE89632 series was submitted by Arendt BM et al. [41] and was developed on the GPL14951 platform, Illumina HumanHT-12 WG-DASL v4.0R2 expression bead chip. Nineteen NAFLD liver biopsies samples (male = 9, female = 10; mean age = 43.47) and twenty-four healthy controls (male = 8, female = 16; mean age = 38.67) were used for the analysis. Patients and healthy controls were recruited from the liver clinic or the Multiorgan Transplant Program, respectively, at the University Health Network, Toronto, Canada. The study was approved by the local Research Ethics Board and followed the guidelines of the 1975 Declaration of Helsinki and its revisions. All participants provided informed written consent. The patient sample was obtained by liver biopsy due to suspicion of NAFLD. The exclusion criteria were: alcohol consumption >20 g/day; any other liver disease; use of medications that could cause steatohepatitis, ursodeoxycholic acid, or any experimental drugs, antioxidants, or PUFA supplements in the 6 months prior to admission; pregnancy or breastfeeding. The samples from the healthy patients were from healthy organs (without steatosis or cirrhosis) that were being evaluated for living donor liver transplantation. The main exclusion criterion was any reason that excluded them from liver donation.

The GSE100927 series was submitted by Steenman M et al. [42], and it was developed on GPL17077 platform Agilent-039494 SurePrint G3 Human GE v2 8 × 60 K Microarray 039,381 (Probe Name version). A total of 69 atherosclerotic tissue samples (male = 57, female = 12; mean age = 70.3) and 35 healthy tissue samples (male = 28, female = 7; mean age = 47.9) were used for the analysis. The atherosclerotic samples were obtained from patients undergoing carotid, femoral, and infrapopliteal endarterectomy; all diseased arteries presented with advanced atherosclerotic plaques. The healthy arteries without atherosclerotic lesions were obtained from organ donation. Written informed consent was obtained from both the patients and next-of-kin donors. Sample collection and handling was carried out according to the guidelines of the Medical and Ethical Committee of Nantes, France. Patients with non-atherosclerotic peripheral arterial disease, thrombosis, or restenosis were excluded.

**Figure 1:**
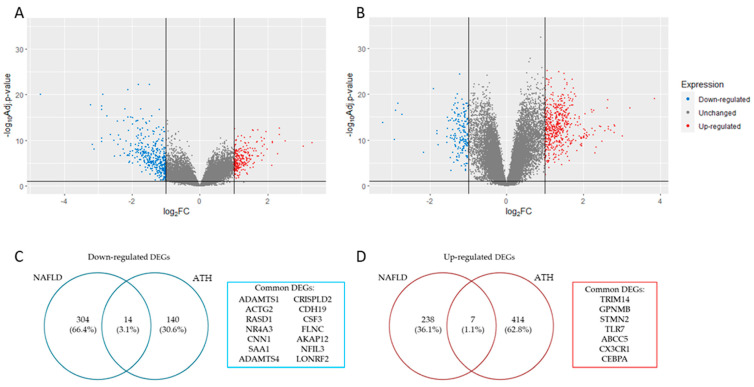
Panels (**A**,**B**) show volcano plot with DEGs identified in NAFLD and ATH, respectively (|log2 FC (fold change)|> 1 and adj. *p*-value < 0.05). Panels (**C**,**D**) show a list with common down- and up-regulated DEGs identified in both pathologies, respectively.

The analysis of the DEGs (|log2 FC (fold change)| > 1 and adj. *p*-value < 0.05) for each gene expression profile resulted in a total of 270 up-regulated and 318 down-regulated genes for NAFLD (Figure 1A), while for ATH, the result was 421 up-regulated genes and 154 down-regulated genes (Figure 1B). The overlapping DEGs that matched in their regulation for both pathologies resulted in 21 genes (14 down-regulated ones (Figure 1C) and 7 up-regulated ones (Figure 1D).

### 2.2. Protein–Protein Interaction (PPI) Network

**Figure 2:**
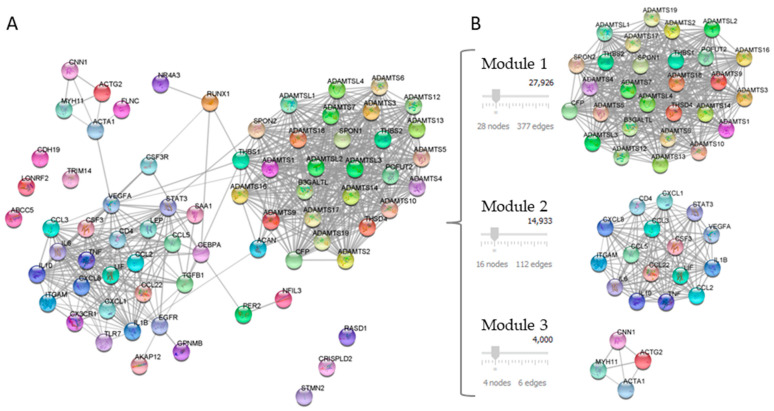
Analysis of the PPI network of DEGs. Panel (**A**) shows PPI network constructed with 21 common proteins between NAFLD and ATH. Panel (**B**) shows the PPI network modules identified using the MCODE plug-in.

Twenty-one DEGs related to NAFLD and ATH were used as a query in the STRING application within the Cytoscape software, with the aim of generate a PPI network. The confidence value of the interacting proteins was set to 0.7, and the maximum additional interactors were 50. The result was a PPI network of 71 nodes with 572 interactions, as shown in Figure 2A.

Seventy-one genes that constituted the PPI network were then used for enrichment analysis, which was performed using the DAVID database. A cutoff point was established for at least 20 genes in order to obtain an overview of the PPI network. The main pathways were involved in o-glycosylation, post-translational protein modification, and cytokine signaling processes. Gene Ontology related them to the extracellular matrix and metallopeptidase activity (Table 1).

### 2.3. Functional Modules and Hubs

To analyze the topology of the constructed PPI, betweenness centrality and degree centrality were calculated. Genes with higher centrality scores were then identified through the CytoNCA. The centrality analysis resulted in the hubs shown in Table 2.

The PPI network modules were identified using the MCODE plug-in and those with a score of above four were selected. The result was the selection of three modules: module 1 contained 28 nodes and 377 edges; module 2 contained 16 nodes and 112 edges, and module 3 contained 4 nodes and 6 edges, which had MCODE scores of 27,926, 14,933, and 4000, respectively (Figure 2B).

In order to further analyze the enrichment of core genes, we conducted a Gene Ontology and REACTOME pathway analysis for the two modules selected in the previous step. The results showed that 28 genes in module 1 were mainly related to O-glycosylation processes and related diseases, which probably affect the metabolism and organization in the extracellular matrix. In this context, metalloendopeptidase activity and the organization of collagen fibers could be involved. Integrin signaling mechanisms and extracellular matrix degradation could be also important.

On the other hand, the genes forming module 2 were mainly associated with Interleukin signaling, which in an immune process that regulated the inflammatory response and cell migration through cytokine signaling. These cytokines could be regulated by mechanisms such as chemotaxis or the regulation of gene expression. In addition, genes belonging to module 2 appear to be linked to protein kinase signaling and growth factors. The negative regulation of cell proliferation also appears to be significant in this module.

Finally, four genes included in module 3 appear to be linked to calcium and ATP binding processes for muscle contraction purposes, but due to the low number of genes included in the module, most of the terms that appeared in the enrichment did not pass the adjusted *p*-value cutoff.

**Figure 3:**
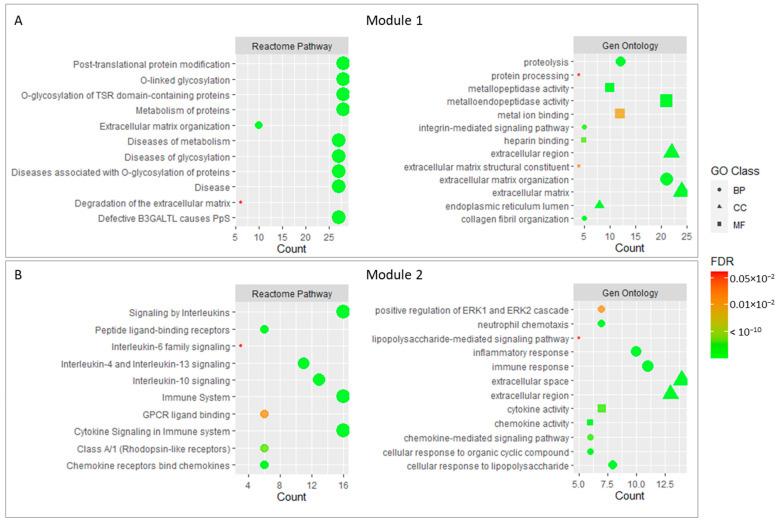
Main enrichment results of module 1 (**A**) and module 2 (**B**). The size of dots corresponds to the number of genes while the color of dots denotes the False Discovery Rate (FDR) value. The shapes of the dots in the Gene Ontology enrichment refer to Biological Process (BP), Molecular Function (MF), and Cellular Component (CC).

Figure 3 shows the main enrichment results for the selected functional modules.

### 2.4. Checking Key Genes through Public Gene–Disease Association Databases

According to the results obtained in the previous sections (centrality analysis and functional module analysis), public gene–disease association databases were used to check the genes of interest. The DEGs common to the two pathologies with certain importance were ADAMTS1 (ADAM metallopeptidase with thrombospondin type 1 Motif 1) and CEBPA (CCAAT Enhancer Binding Protein Alpha), as genes with high centrality scores, and as for the functional module analysis, they were ADAMTS1 and ADAMTS4 for module 1 and CSF3 (Colony-Stimulating Factor 3) for module 2.

As shown in Table 3, the genes selected for screening in the gene–disease association databases showed a strong association with ATH and NAFLD pathologies (or very similar pathologies). The two metallopeptidases ADAMTS1 and ADAMTS4 were related to ATH in practically all of the databases we consulted, while their relationship with the liver was shared between oncologic processes and fatty liver. On the other hand, the genes CEBPA and CSF3 appeared to be closely related to NAFLD and cardiovascular processes, including ATH.

## 3. Discussion

Currently, the relationship between diseases of the cardiovascular system and liver disease is poorly explored. Despite this, studies connecting these types of diseases are gradually appearing. The studies linking ATH and NAFLD previously performed by other authors motivated us to perform this study exclusively using bioinformatics tools. The previous studies have suggested a connection between the two diseases through oxidative stress processes, inflammatory processes, coagulation factors, and hepatokine involvement, which would indicate an overlap in the molecular mechanisms shared by ATH and NAFLD [17,18].

The results of this study show that by using the genetic data submitted by Arendt BM et al. [41] and by Steenman M et al. [42] for NAFLD and ATH processes, respectively, there is a total of 21 common DEGs shared in both pathologies, of which 14 genes were down-regulated and 7 genes were up-regulated. Among them, four genes were selected as especially relevant to the NAFLD/ATH axis due their high score in the centrality analyses or due their involvement in the functional modules identified in this study. Thus, ADAMTS1, ADAMTS4, CEBPA, and CSF3 were proposed as common genes between both pathologies.

Regarding ADAMTS1, it is a metalloproteinase belonging to the ADAMS family, which is involved in extracellular matrix (ECM) remodeling, a process regulated by a variety of modifiers, including enhancers and inhibitors [43]. This protein plays an important role in degrading ECM components and inhibiting angiogenesis [44,45,46,47] via its metalloprotease-dependent catalytic and thrombospondin-dependent regions [47,48,49]. In this context, it participates in the degradation of pro-collagen, proteoglycans, and the cartilage oligomeric matrix protein [50,51]. Moreover, the knockdown of ADAMTS1 seems to promote cell migration [52], which could be related to ATH development [53].

On the other hand, the down-regulation of ADMTS1 has been associated with Metabolic Dysfunction-Associated Fatty Liver Disease (MAFLD) [54]. In relation to this, it has recently been shown that the inhibition of ADAMTS1 in adipose tissue leads to adipose tissue expansion, together with decreased insulin sensitivity and dysfunctional lipid metabolism [43,54], and thus, appears to contribute to the maintenance of lipid homeostasis [54]. These findings are consistent with the decreased expression of this protein in the adipose tissue of obese mice and with an inverse correlation of ADAMTS1 expression with body mass index in humans [43].

In exploring the link between NAFLD and ATH, ADAMTS1 may be involved in both pathologies through the metalloproteinase MMP1 signaling pathway. TFPI-2, a factor associated with ECM remodeling and ATH [55,56], has been identified as a binding partner for ADAMTS1 by Torres-Collado, A. et al. [43]. TFPI-2 participates in the inhibition of matrix metalloproteinases MMP-1 [56], which is involved in the degradation of type I and III fibrillar collagens and the matrix proteins, which are the main components of the endothelial and subendothelial walls [57,58]. The degradation of these proteins allows the migration and subsequent expansion of leukocytes and VSMCs [59], leading to the development of atheroma plaques and the thickening of the intima-media layer [60,61]. Furthermore, MMP1 was found to be expressed in monocytes, Kupffer cells, and liver stellate cells early in the development of non-alcoholic steatohepatitis (NASH) [62,63], as well as in the hepatocyte progenitor cells participating in the process of angiogenesis in advanced NASH [63]. Therefore, the down-regulation of ADAMTS1 observed in our study could be related to ATH and NAFLD, leading to a decrease in TFPI-2 activity and overexpression of MMP1. In addition, ADAMTS1, which was also identified as an inflammatory associated protein, is required for a balanced immune response [64,65]. It is known that both innate and adaptive immune systems are involved in NAFLD pathogenesis, and crosstalk between the immune cells and liver cells participates in its initiation and progression [66]. In the case of ATH, the involvement of the immune system in its development is well known [67]. In accordance with these studies, the decreased levels of ADAMTS1 observed in our analysis from the NAFLD and ATH samples could be related to an abnormal immune response, contributing to the development of both disorders. Accordingly, the PPI network enrichment analysis performed in this study already showed a link to the cytokine-related processes, immune system processes and protein modifications. These results suggest that the role of ADAMTS1 in the NAFLD/ATH axis could be explained by its participation in different signaling pathways.

However, the exact role of ADAMTS1 it is not completely understood, since there are some studies that demonstrate opposite results, showing a link between ADAMTS1 and the development of atherosclerotic plaques and ATH [68]. Moreover, ADAMTS1 has been found to be overexpressed in the intima of atherosclerotic plaques [45,69,70], as well as in the neutrophils and macrophages accumulated in the aortic tissues of patients with acute aortic dissection [71]. Regarding liver diseases, ADAMTS1 has been associated with the ability to activate TGF-b in liver fibrosis [72,73], as well as with NASH [74]. Due to the controversy shown regarding the role of ADAMTS1 in the development of ATH and NAFLD, further studies are needed to elucidate the real role of this protein.

Regarding ADAM metallopeptidase with thrombospondin type 1 motif 4 (ADAMTS4), it is an important analog of ADAMTS1. Studies have shown that several inflammation-associated signals also reduce ADAMTS4 expression, leading to a subsequent increased accumulation of ECM components, which could contribute to the fibrotic deposition of collagen [75]. In this way, ADAMTS4 also appears to be downregulated in our functional module results, which could be related to the development of NAFLD and ATH.

Regarding CEBPA or CEBPα, this protein is one of the factors that regulate the process of adipogenesis, together with PPARγ, and is involved in the sequential expression of adipocyte-specific proteins [76,77,78,79,80,81,82]. It is also essential for the myeloid lineage maturation process [83]. CEBPA appears to be expressed in inflammatory processes [83], although its exact function is unknown. Zhou J et al. observed, in 2019, that its overexpression increases the neutrophil population in a murine model [84]. Neutrophils are well known to respond to acute inflammation, but they are also linked to chronic inflammation [85,86]. Recent studies have related neutrophils to the formation of neutrophil extracellular traps (NETs) through a process called NETosis [86,87,88], which can promote the inflammatory process by stimulating the synthesis of ROS and proinflammatory cytokines by macrophages [89]. These NETs have been found in atherogenic plaques in both murine and human models [90,91,92], and the inhibition of NETosis has been linked to a decrease in the size of atherogenic plaques and an increase in plaque instability. Therefore, an overexpression of CEBPA may contribute to the development of ATH, thus promoting the increase in the neutrophil populations and the formation of those NETs.

On the other hand, Bristol J.A et al. observed that CEBPA, together with CEBPB, binds to the TNFR1 promoter, increasing its expression and inducing an increase in TNF expression through a positive feedback mechanism [93]. TNFα is a known factor to promote the development of ATH and NAFLD [29,94,95,96], so CEBPA may contribute to these diseases through the TNFα pathway.

In accordance with this, our results show CEBPA overexpression in both the NAFLD and ATH samples. This protein appears in the module 2, which is closely related to interleukin signaling, immune response, and cytokine activity. However, further studies are needed to better understand the specific role of this protein, since some studies suggest its relationship with anti-inflammatory process in murine models [84,97], which are in contrast to the studies mentioned above. Currently, there is a very little amount of information available about this protein, so bioinformatics studies such as the one we have carried out are very valuable as they are able to identify interesting potential biomarkers that are unexplored to date. In this sense, it seems promising to intensively study the mechanism of action of CEBPA to explore its potential role as a biomarker of NAFLD and ATH or as a possible therapeutic target.

Another gene of interest identified in our study is the CSF3 gene, which encodes a member of the IL-6 superfamily of cytokines. The encoded cytokine controls the production, differentiation, and function of granulocytes. The importance of this gene in NAFLD is described by Nam et al., in which they demonstrate that a treatment with CSF3 in animal models had a possible protective effect by reducing hepatocyte apoptosis and by increasing cell survival and the anti-inflammatory function [98]. Regarding ATH, it has been shown that CSF3 therapy inhibits the atherosclerotic process in animal models [99]. In our study, the CSF3 gene does not appear with a high score in the centrality analysis, but it does appear as a member of the second functional module identified. According to the results of DEGs analysis, CSF3 appears to be down-regulated in our samples for both ATH and NAFLD, which agrees with the results of the published studies, being that this under-expression of CSF3 the possible cause of the development of NAFLD and ATH.

Our study has some limitations, such as the reduced number of samples used to compare, which is mainly due to the low number of public data series with an adequate level of quality or information and to the lack of control datasets that can be used to compare the pathological samples with healthy samples. Another important limitation would be the exclusion of potential targets involved in the NAFLD/ATH axis in the bioinformatics analysis due to establishment of a specific cutoff point. However, the enrichment analysis performed negates this limitation, avoiding the loss of potential candidates. Additionally, expression studies at the proteomic level would be useful to validate the obtained results since only genetic data and bioinformatic studies have been analyzed. The strength of our study lies in the generation of a strategy that is capable of combining and jointly exploiting the information available through different bioinformatic tools, generating very valuable information for the identification of new potential targets related to these highly prevalent pathologies. More experimental studies are necessary to understand the specific role of the identified proteins in NAFLD and ATH, however, the first step is to identify some potential good candidates to explore as biomarkers or therapeutic targets of these disorders.

In summary, our findings suggest that atherosclerotic processes could share common molecular pathways with the development of some liver disorders such as NAFLD. Our study identified some novel potential targets in the NAFLD/ATH axis, including ADAMTS1, ADAMTS, CEBPA, and CSF3, using mainly bioinformatics tools. Considering that cardiovascular disease (including ATH) is the leading cause of death worldwide and has a major socioeconomic and health care impact and NAFLD has an increasingly higher incidence and is the most prevalent liver disease, affecting 70% of diabetic or obese patients, research into the common molecules involved in the development of these highly prevalent pathologies can have a great impact on clinical practice. The potential role of these molecules as early biomarkers of NAFLD and ATH could contribute to the development of preventive tools, with the aim of avoiding the appearance of irreversible complications in affected patients. On the other hand, as seen in the aforementioned studies, the modulation of these molecules could be used as therapeutic strategies, slowing down or improving the symptomatology of these diseases. Although future experimental studies are needed to confirm the dual function of these proteins in both pathologies, this study provides valuable information for the study of the utility of these proteins as potential biomarkers or therapeutic targets, which could improve the quality of life of affected patients.

## 4. Materials and Methods

### 4.1. Data Acquisition and Visualization and Identification of Differentially Expressed Genes

The NCBI-GEO is a public functional genomics database repository. By searching for keywords, such as ATH NAFLD in *Homo sapiens*, two series were selected that could be used to compare the differential genes of both pathologies.

To obtain the differentially expressed genes between the healthy and pathological samples of both pathologies, we used GEO2R. GEO2R is an interactive web-based tool that allows the users to compare datasets in the GEO series to determine the DEGs. |log2 FC (fold change)| > 1 and adj. *p*-value < 0.05 were considered to be statistically significant. DEGs with a log2 FC ≤ −1 or a log2 FC ≥ 1 were considered to be down-regulated and up-regulated, respectively. The Benjamini–Hochberg False Discovery Rate (FDR) was used for *p*-value correction.

### 4.2. Protein–Protein Interaction (PPI) Network

To understand the interactions among the common down-regulated and up-regulated DEGs, the Cytoscape software [100] was used to analyze and visualize the biological network of interaction. Cytoscape provides an open source environment for the large-scale integration of molecular interaction network data. In addition, Cytoscape enables integration with stringApp [101] to facilitate the visualization of network data from the STRING database [102]. To provide more robustness to the analysis, the 50 nearest interactors to the identified SDRs were included. The confidence score (cutoff) was set to 0.7 (high confidence).

Functional annotation was performed with the Database for Annotation, Visualization, and Integrated Discovery (DAVID) [103].

### 4.3. Functional Modules and Hubs

Subsequently, CytoNCA [104], a Cytoscape plugin was used to perform a centrality analysis and identify essential proteins within the biological network. CytoNCA calculated the node scores by applying two centrality methods: degree centrality (defined as the number of links incident upon a node) and betweenness centrality (defined as the amount of influence a node has on the flow of information in a network).

In addition, the main functional modules were analyzed using Cytoscape’s Molecular Complex Detection (MCODE) plug-in [105]. MCODE was used to perform the graph-theoretic clustering to detect dense regions of protein–protein interaction networks based on connectivity data, most of which correspond to known protein complexes. The parameters set for screening the function were as follows: degree cutoff = 2, max depth = 100, k-score = 2, and node score cutoff = 0.2. Only modules with an MCODE score of at least 4 were selected. A new functional enrichment analysis using DAVID was performed on those modules with the best scores.

### 4.4. Checking Key Genes through Public Gene–Disease Association Databases

The DEGs resulting from the above analyses, i.e., those that scored highly in the centrality tests and were part of identified functional modules, were validated by text-mining using databases such as DisGeNET, MalaCards, and HuGE Genopedia.

The DisGeNET database was used to obtain the genes associated with ATH and NAFLD. DisGeNET is a discovery platform containing one of the largest publicly available collections of genes and variants associated with human diseases [106]. The latest update available is version 7 (June 2020) containing 1,134,942 gene–disease associations (GDAs) between 21,671 genes and 30,170 diseases and traits. The data contained in this database come from the most popular repositories used by the scientific community. In addition, these data are expanded and enriched with information extracted from scientific literature using state-of-the-art text-mining tools.

MalaCards is an integrated database of human pathologies and their annotations. This database is organized into disease cards containing information, annotations, connections between other diseases, as well as genes associated with each disease. It currently contains 22,091 disease entries, which come from 75 sources [107].

HuGE Genopedia is a database that focuses on genetic association studies summarized in Human Genome Epidemiology (HuGE). Following its latest available data update, it contained 16,498 genes and 3416 diseases. Using a single gene as a query, it provides summary information on diseases that have been studied in association with the given query [108].

## 5. Conclusions

Scientific evidence suggests that the development of atherosclerotic processes may share molecular mechanisms with the development of NAFLD. Supporting this evidence, our results indicated two main targets that were highlighted as hubs in the bioinformatics analyses that we carried out: ADAMST1 and CEBPA. However, additional targets could be considered, although with a lower score than those of the two mentioned proteins. This molecular relationship between both pathologies opens the door to the design of therapeutic strategies that can contribute to the improvement of the quality of life of affected patients or even to the development of preventive strategies for use by the population at a higher risk of suffering from these complications.

## Figures and Tables

**Table 1:** Functional enrichment of the PPI network.

Category	ID	Term	Count	*p*-Value	FDR
Reactome pathway	R-HSA-5173214	O-glycosylation of TSR domain-containing proteins	28	2.78 × 10^−54^	9.50 × 10^−52^
Reactome pathway	R-HSA-5083635	Defective B3GALTL causes PpS	27	5.38 × 10^−52^	9.20 × 10^−50^
Reactome pathway	R-HSA-3906995	Diseases associated with O-glycosylation of proteins	27	2.46 × 10^−42^	2.80 × 10^−40^
Reactome pathway	R-HSA-5173105	O-linked glycosylation	28	8.67 × 10^−38^	7.42 × 10^−36^
Reactome pathway	R-HSA-3781865	Diseases of glycosylation	28	1.65 × 10^−34^	1.13 × 10^−32^
GO-Term CC	GO:0031012	extracellular matrix	28	1.54 × 10^−33^	1.84 × 10^−31^
GO-Term BP	GO:0030198	extracellular matrix organization	23	4.30 × 10^−29^	4.57 × 10^−26^
Reactome pathway	R-HSA-5668914	Diseases of metabolism	28	1.14 × 10^−27^	6.50 × 10^−26^
GO-Term MF	GO:0004222	metalloendopeptidase activity	21	1.46 × 10^−27^	2.55 × 10^−25^
GO-Term CC	GO:0005576	Extracellular region	41	6.39 × 10^−22^	3.80 × 10^−20^
Reactome pathway	R-HSA-1643685	Disease	38	8.84 × 10^−14^	3.78 × 10^−12^
GO-Term CC	GO:0005615	Extracellular space	28	5.48 × 10^−11^	2.17 × 10^−9^
Reactome pathway	R-HSA-597592	Post-translational protein modification	30	7.31 × 10^−10^	2.27 × 10^−8^
Reactome pathway	R-HSA-392499	Metabolism of proteins	33	1.38 × 10^−8^	3.92 × 10^−7^
Reactome pathway	R-HSA-1280215	Cytokine Signaling in Immune system	20	3.16 × 10^−8^	8.32 × 10^−7^
Reactome pathway	R-HSA-168256	Immune System	23	2.81 × 10^−3^	5.33 × 10^−2^
Reactome pathway	R-HSA-162582	Signal Transduction	22	8.14 × 10^−2^	7.52 × 10^−1^
Reactome pathway	R-HSA-5173214	O-glycosylation of TSR domain-containing proteins	28	2.78 × 10^−54^	9.50 × 10^−52^
Reactome pathway	R-HSA-5083635	Defective B3GALTL causes PpS	27	5.38 × 10^−52^	9.20 × 10^−50^
Reactome pathway	R-HSA-3906995	Diseases associated with O-glycosylation of proteins	27	2.46 × 10^−42^	2.80 × 10^−40^

Count: count of genes in the PPI network that share the same term; FDR: False Discovery Rate; GO-Term: Gene Ontology term; BP: biological process; MF: molecular function; CC: cellular component.

**Table 2:** PPI network centrality ranking.

Ranking	Gen Symbol	Uniprot:ID *	Description	Betweenness	Degree
1	VEGFA	Q9H1W9	Vascular endothelial growth factor A	1208,552	19.0
2	THBS1	P07996	Thrombospondin 1	969,255	29.0
3	ACTA1	P68133	Actin alpha 1	478,000	5.0
4	ADAMTS1	Q9UHI8	ADAM Metallopeptidase with Thrombospondin Type 1 Motif 1	459,444	29.0
5	SPON2	Q9BUD6	Spondin 2	314,440	28.0
6	ITGAM	P11215	Integrin Subunit Alpha M	304,704	14.0
7	CEBPA	P49715	CCAAT Enhancer Binding Protein Alpha	292,120	8.0
8	TGFB1	P01137	Transforming Growth Factor Beta 1	287,228	12.0
9	RUNX1	Q16285	RUNX Family Transcription Factor 1	256,626	4.0
10	EGFR	Q9H2C9	Epidermal Growth Factor Receptor	247,008	11.0

* Uniprot Identifier.

**Table 3:** Diseases related to genes common to ATH and NAFLD with high scores in centrality analysis or included in the functional modules of interest.

Gen	Group	Disgenet	MalaCards	HuGE Genopedia
ADAMTS1	Liver-related diseases	Liver Carcinoma	Liver Cirrhosis	Fatty Liver
		Fibrosis, Liver		
	Cardiovascular Diseases	Atherosclerosis	Aortic Aneurysm	Coronary Disease
		Arteriosclerosis	Atherosclerosis Susceptibility	Cardiovascular Diseases
		Coronary Arteriosclerosis	Ischemia	
		Coronary Artery Disease		
ADAMTS4	Liver-related diseases	Liver Carcinoma	Hepatocellular Carcinoma	
	Cardiovascular Diseases	Atherosclerosis	Cerebrovascular Disease	Aortic Aneurysm, Thoracic
		Arteriosclerosis	Cardiovascular System Disease	
		Aortic Aneurysm	Carotid Stenosis	
		Coronary Arteriosclerosis	Atherosclerosis Susceptibility	
CEBPA	Liver-related diseases	Liver Carcinoma	NAFLD	Liver Neoplasms
		NAFLD	Fatty Liver Disease	
		Fatty liver	Liver Cirrhosis	
	Cardiovascular Diseases	Atherosclerosis	Cerebral Artery Occlusion	Myocardial Ischemia
		Arteriosclerosis	Peripheral Artery Disease	Peripheral Vascular Diseases
		Cardiovascular Diseases	Ischemia	
		Peripheral Arterial Diseases		
CSF3	Liver-related diseases	Fatty Liver Disease	NAFLD	Liver Cirrhosis
		NAFLD	Fatty Liver Disease	
		Liver Carcinoma	Liver Disease	
		Acute-On-Chronic Liver Failure	Liver Cirrhosis	
	Cardiovascular Diseases	Atherosclerosis	Carotid Artery Disease	Cardiovascular Diseases
		Arteriosclerosis	Ischemia	Carotid Stenosis
		Arteriosclerosis Obliterans	Vascular Disease	Coronary Artery Disease
		Ischemic cardiomyopathy	Hepatic Vascular Disease	
		Coronary Arteriorclerosis		

## Data Availability

The data corresponding to microarray expression profile datasets GSE89632 and GSE100927 presented in this study are openly available at https://www.ncbi.nlm.nih.gov/geo/query/acc.cgi?acc=GSE89632, (accessed on 3 January 2023) and https://www.ncbi.nlm.nih.gov/geo/query/acc.cgi?acc=GSE100927 (accessed on 3 January 2023), respectively. Series GSE89632 was submitted by Arendt BM et al. [41] and series GSE100927 by Steenman M et al. [42].

## References

[1] Li H., Yu X.-H., Ou X., Ouyang X.-P., Tang C.-K. (2021). Hepatic Cholesterol Transport and Its Role in Non-Alcoholic Fatty Liver Disease and Atherosclerosis. Prog. Lipid Res..

[2] Chalasani N., Younossi Z., Lavine J.E., Diehl A.M., Brunt E.M., Cusi K., Charlton M., Sanyal A.J., American Gastroenterological Association, American Association for the Study of Liver Diseases (2012). The Diagnosis and Management of Non-Alcoholic Fatty Liver Disease: Practice Guideline by the American Gastroenterological Association, American Association for the Study of Liver Diseases, and American College of Gastroenterology. Gastroenterology.

[3] Farrell G.C., Larter C.Z. (2006). Nonalcoholic Fatty Liver Disease: From Steatosis to Cirrhosis. Hepatology.

[4] Ampuero J., Gallego-Durán R., Romero-Gómez M. (2015). Association of NAFLD with Subclinical Atherosclerosis and Coronary-Artery Disease: Meta-Analysis. Rev. Esp. Enferm. Dig..

[5] Angulo P. (2002). Nonalcoholic Fatty Liver Disease. N. Engl. J. Med..

[6] Clouston A.D., Powell E.E. (2004). Nonalcoholic Fatty Liver Disease: Is All the Fat Bad?. Intern. Med. J..

[7] Gofton C., Upendran Y., Zheng M.-H., George J. (2022). MAFLD: What Is Different from NAFLD?. Clin. Mol. Hepatol..

[8] Hassen G., Singh A., Belete G., Jain N., De la Hoz I., Camacho-Leon G.P., Dargie N.K., Carrera K.G., Alemu T., Jhaveri S. (2022). Nonalcoholic Fatty Liver Disease: An Emerging Modern-Day Risk Factor for Cardiovascular Disease. Cureus.

[9] Cai J., Zhang X.-J., Ji Y.-X., Zhang P., She Z.-G., Li H. (2020). Nonalcoholic Fatty Liver Disease Pandemic Fuels the Upsurge in Cardiovascular Diseases. Circ. Res..

[10] Stahl E.P., Dhindsa D.S., Lee S.K., Sandesara P.B., Chalasani N.P., Sperling L.S. (2019). Nonalcoholic Fatty Liver Disease and the Heart: JACC State-of-the-Art Review. J. Am. Coll. Cardiol..

[11] Lee H., Lee Y.-H., Kim S.U., Kim H.C. (2021). Metabolic Dysfunction-Associated Fatty Liver Disease and Incident Cardiovascular Disease Risk: A Nationwide Cohort Study. Clin. Gastroenterol. Hepatol. Off. Clin. Pract. J. Am. Gastroenterol. Assoc..

[12] Salgado Alvarez G.A., Pinto Galvez S.M., Garcia Mora U., Cano Contreras A.D., Durán Rosas C., Priego-Parra B.A., Triana Romero A., Amieva Balmori M., Roesch Dietlen F., Martinez Vazquez S.E. (2022). Higher Cardiovascular Risk Scores and Liver Fibrosis Risk Estimated by Biomarkers in Patients with Metabolic-Dysfunction-Associated Fatty Liver Disease. World J. Hepatol..

[13] Hoebinger C., Rajcic D., Hendrikx T. (2021). Oxidized Lipids: Common Immunogenic Drivers of Non-Alcoholic Fatty Liver Disease and Atherosclerosis. Front. Cardiovasc. Med..

[14] Glass C.K., Witztum J.L. (2001). Atherosclerosis. the Road Ahead. Cell.

[15] Ross R. (1999). Atherosclerosis—An Inflammatory Disease. N. Engl. J. Med..

[16] World Health Organization (WHO). https://www.who.int.

[17] Zhang L., She Z.-G., Li H., Zhang X.-J. (2020). Non-Alcoholic Fatty Liver Disease: A Metabolic Burden Promoting Atherosclerosis. Clin. Sci..

[18] Gaudio E., Nobili V., Franchitto A., Onori P., Carpino G. (2012). Nonalcoholic Fatty Liver Disease and Atherosclerosis. Intern. Emerg. Med..

[19] Abdallah L.R., de Matos R.C., E Souza Y.P.D.M., Vieira-Soares D., Muller-Machado G., Pollo-Flores P. (2020). Non-Alcoholic Fatty Liver Disease and Its Links with Inflammation and Atherosclerosis. Curr. Atheroscler. Rep..

[20] Choe Y.G., Jin W., Cho Y.K., Chung W.G., Kim H.J., Jeon W.K., Kim B.I. (2013). Apolipoprotein B/AI Ratio Is Independently Associated with Non-Alcoholic Fatty Liver Disease in Nondiabetic Subjects. J. Gastroenterol. Hepatol..

[21] Klop B., Elte J.W.F., Cabezas M.C. (2013). Dyslipidemia in Obesity: Mechanisms and Potential Targets. Nutrients.

[22] Nikolic D., Katsiki N., Montalto G., Isenovic E.R., Mikhailidis D.P., Rizzo M. (2013). Lipoprotein Subfractions in Metabolic Syndrome and Obesity: Clinical Significance and Therapeutic Approaches. Nutrients.

[23] Davies B.S.J., Beigneux A.P., Barnes R.H., Tu Y., Gin P., Weinstein M.M., Nobumori C., Nyrén R., Goldberg I., Olivecrona G. (2010). GPIHBP1 Is Responsible for the Entry of Lipoprotein Lipase into Capillaries. Cell Metab..

[24] Atef M.E., Anand-Srivastava M.B. (2016). Role of PKCδ in Enhanced Expression of Gqα/PLCβ1 Proteins and VSMC Hypertrophy in Spontaneously Hypertensive Rats. PLoS ONE.

[25] McLean K., Schirm S., Johns A., Morser J., Light D.R. (2001). FXa-Induced Responses in Vascular Wall Cells Are PAR-Mediated and Inhibited by ZK-807834. Thromb. Res..

[26] Koch M., Zernecke A. (2014). The Hemostatic System as a Regulator of Inflammation in Atherosclerosis. IUBMB Life.

[27] Marin V., Farnarier C., Grès S., Kaplanski S., Su M.S., Dinarello C.A., Kaplanski G. (2001). The P38 Mitogen-Activated Protein Kinase Pathway Plays a Critical Role in Thrombin-Induced Endothelial Chemokine Production and Leukocyte Recruitment. Blood.

[28] Shu B., Yang Y., Qian M. (2014). The phenotypic switching of vascular smooth muscle cells induced by cholesterol. Xi Bao Yu Fen Zi Mian Yi Xue Za Zhi.

[29] Chistiakov D.A., Orekhov A.N., Bobryshev Y.V. (2015). Vascular Smooth Muscle Cell in Atherosclerosis. Acta Physiol..

[30] Provost P., Lam J.Y., Lacoste L., Merhi Y., Waters D. (1994). Endothelium-Derived Nitric Oxide Attenuates Neutrophil Adhesion to Endothelium under Arterial Flow Conditions. Arterioscler. Thromb..

[31] Palmer R.M., Ferrige A.G., Moncada S. (1987). Nitric Oxide Release Accounts for the Biological Activity of Endothelium-Derived Relaxing Factor. Nature.

[32] Wang X., Wang W., Li Y., Bai Y., Fiscus R.R. (1999). Mechanism of SNAP Potentiating Antiproliferative Effect of Calcitonin Gene-Related Peptide in Cultured Vascular Smooth Muscle Cells. J. Mol. Cell. Cardiol..

[33] Kietadisorn R., Juni R.P., Moens A.L. (2012). Tackling Endothelial Dysfunction by Modulating NOS Uncoupling: New Insights into Its Pathogenesis and Therapeutic Possibilities. Am. J. Physiol. Endocrinol. Metab..

[34] Rekka E.A., Chrysselis M.C. (2002). Nitric Oxide in Atherosclerosis. Mini Rev. Med. Chem..

[35] Gimbrone M.A., García-Cardeña G. (2016). Endothelial Cell Dysfunction and the Pathobiology of Atherosclerosis. Circ. Res..

[36] Stary H.C. (2000). Natural History and Histological Classification of Atherosclerotic Lesions: An Update. Arterioscler. Thromb Vasc. Biol..

[37] Pan Y., Wang Y., Xu J., Wu J., Chen Q., Zeng G., Zhao G. (2017). TG and VLDL Cholesterol Activate NLRP1 Inflammasome by Nuclear Factor-ΚB in Endothelial Cells. Int. J. Cardiol..

[38] Kawakami A., Aikawa M., Alcaide P., Luscinskas F.W., Libby P., Sacks F.M. (2006). Apolipoprotein CIII Induces Expression of Vascular Cell Adhesion Molecule-1 in Vascular Endothelial Cells and Increases Adhesion of Monocytic Cells. Circulation.

[39] Bisgaard L.S., Mogensen C.K., Rosendahl A., Cucak H., Nielsen L.B., Rasmussen S.E., Pedersen T.X. (2016). Bone Marrow-Derived and Peritoneal Macrophages Have Different Inflammatory Response to OxLDL and M1/M2 Marker Expression—Implications for Atherosclerosis Research. Sci. Rep..

[40] Yan J., Stringer S.E., Hamilton A., Charlton-Menys V., Götting C., Müller B., Aeschlimann D., Alexander M.Y. (2011). Decorin GAG Synthesis and TGF-β Signaling Mediate Ox-LDL-Induced Mineralization of Human Vascular Smooth Muscle Cells. Arterioscler. Thromb. Vasc. Biol..

[41] Arendt B.M., Comelli E.M., Ma D.W.L., Lou W., Teterina A., Kim T., Fung S.K., Wong D.K.H., McGilvray I., Fischer S.E. (2015). Altered Hepatic Gene Expression in Nonalcoholic Fatty Liver Disease Is Associated with Lower Hepatic n-3 and n-6 Polyunsaturated Fatty Acids. Hepatology.

[42] Steenman M., Espitia O., Maurel B., Guyomarch B., Heymann M.-F., Pistorius M.-A., Ory B., Heymann D., Houlgatte R., Gouëffic Y. (2018). Identification of Genomic Differences among Peripheral Arterial Beds in Atherosclerotic and Healthy Arteries. Sci. Rep..

[43] Chen S.-Z., Ning L.-F., Xu X., Jiang W.-Y., Xing C., Jia W.-P., Chen X.-L., Tang Q.-Q., Huang H.-Y. (2016). The MiR-181d-Regulated Metalloproteinase Adamts1 Enzymatically Impairs Adipogenesis via ECM Remodeling. Cell Death Differ..

[44] Jones G.C., Riley G.P. (2005). ADAMTS Proteinases: A Multi-Domain, Multi-Functional Family with Roles in Extracellular Matrix Turnover and Arthritis. Arthritis Res. Ther..

[45] Salter R.C., Ashlin T.G., Kwan A.P.L., Ramji D.P. (2010). ADAMTS Proteases: Key Roles in Atherosclerosis?. J. Mol. Med..

[46] Ashlin T.G., Kwan A.P.L., Ramji D.P. (2013). Regulation of ADAMTS-1, -4 and -5 Expression in Human Macrophages: Differential Regulation by Key Cytokines Implicated in Atherosclerosis and Novel Synergism between TL1A and IL-17. Cytokine.

[47] Rodríguez-Manzaneque J.C., Carpizo D., Plaza-Calonge M. (2009). del C.; Torres-Collado, A.X.; Thai, S.N.-M.; Simons, M.; Horowitz, A.; Iruela-Arispe, M.L. Cleavage of Syndecan-4 by ADAMTS1 Provokes Defects in Adhesion. Int. J. Biochem. Cell Biol..

[48] Esselens C., Malapeira J., Colomé N., Casal C., Rodríguez-Manzaneque J.C., Canals F., Arribas J. (2010). The Cleavage of Semaphorin 3C Induced by ADAMTS1 Promotes Cell Migration. J. Biol. Chem..

[49] Luque A., Carpizo D.R., Iruela-Arispe M.L. (2003). ADAMTS1/METH1 Inhibits Endothelial Cell Proliferation by Direct Binding and Sequestration of VEGF165. J. Biol. Chem..

[50] Kuno K., Okada Y., Kawashima H., Nakamura H., Miyasaka M., Ohno H., Matsushima K. (2000). ADAMTS-1 Cleaves a Cartilage Proteoglycan, Aggrecan. FEBS Lett..

[51] Li M., Liu Q., Lei J., Wang X., Chen X., Ding Y. (2017). MiR-362-3p Inhibits the Proliferation and Migration of Vascular Smooth Muscle Cells in Atherosclerosis by Targeting ADAMTS1. Biochem. Biophys. Res. Commun..

[52] Lambert J., Makin K., Akbareian S., Johnson R., Alghamdi A.A.A., Robinson S.D., Edwards D.R. (2020). ADAMTS-1 and Syndecan-4 Intersect in the Regulation of Cell Migration and Angiogenesis. J. Cell Sci..

[53] Rudijanto A. (2007). The Role of Vascular Smooth Muscle Cells on the Pathogenesis of Atherosclerosis. Acta Med. Indones..

[54] Xiao C., Chen S., Yang C., Liu J., Yu M. (2022). Identification of Polyunsaturated Fatty Acids Related Key Modules and Genes in Metabolic Dysfunction-Associated Fatty Liver Disease Using WGCNA Analysis. Front. Genet..

[55] Torres-Collado A.X., Kisiel W., Iruela-Arispe M.L., Rodríguez-Manzaneque J.C. (2006). ADAMTS1 Interacts with, Cleaves, and Modifies the Extracellular Location of the Matrix Inhibitor Tissue Factor Pathway Inhibitor-2. J. Biol. Chem..

[56] Herman M.P., Sukhova G.K., Kisiel W., Foster D., Kehry M.R., Libby P., Schönbeck U. (2001). Tissue Factor Pathway Inhibitor-2 Is a Novel Inhibitor of Matrix Metalloproteinases with Implications for Atherosclerosis. J. Clin. Investig..

[57] Kaysen G.A., Eiserich J.P. (2004). The Role of Oxidative Stress-Altered Lipoprotein Structure and Function and Microinflammation on Cardiovascular Risk in Patients with Minor Renal Dysfunction. J. Am. Soc. Nephrol..

[58] Südhof T.C., Goldstein J.L., Brown M.S., Russell D.W. (1985). The LDL Receptor Gene: A Mosaic of Exons Shared with Different Proteins. Science.

[59] Kang H.M., Ahn S.H., Choi P., Ko Y.-A., Han S.H., Chinga F., Park A.S.D., Tao J., Sharma K., Pullman J. (2015). Defective Fatty Acid Oxidation in Renal Tubular Epithelial Cells Has a Key Role in Kidney Fibrosis Development. Nat. Med..

[60] Brown A.J., Sun L., Feramisco J.D., Brown M.S., Goldstein J.L. (2002). Cholesterol Addition to ER Membranes Alters Conformation of SCAP, the SREBP Escort Protein That Regulates Cholesterol Metabolism. Mol. Cell.

[61] Radhakrishnan A., Sun L.-P., Kwon H.J., Brown M.S., Goldstein J.L. (2004). Direct Binding of Cholesterol to the Purified Membrane Region of SCAP: Mechanism for a Sterol-Sensing Domain. Mol. Cell.

[62] Ando W., Yokomori H., Tsutsui N., Yamanouchi E., Suzuki Y., Oda M., Inagaki Y., Otori K., Okazaki I. (2018). Serum Matrix Metalloproteinase-1 Level Represents Disease Activity as Opposed to Fibrosis in Patients with Histologically Proven Nonalcoholic Steatohepatitis. Clin. Mol. Hepatol..

[63] Yokomori H., Oda M., Ando W., Inagaki Y., Okazaki I. (2017). Hepatic Progenitor Cell Expansion in Early-Stage Nonalcoholic Steatohepatitis: Evidence from Immunohistochemistry and Immunoelectron Microscopy of Matrix Metalloproteinase-1. Med. Mol. Morphol..

[64] Kuno K., Kanada N., Nakashima E., Fujiki F., Ichimura F., Matsushima K. (1997). Molecular Cloning of a Gene Encoding a New Type of Metalloproteinase-Disintegrin Family Protein with Thrombospondin Motifs as an Inflammation Associated Gene. J. Biol. Chem..

[65] Rodríguez-Baena F.J., Redondo-García S., Peris-Torres C., Martino-Echarri E., Fernández-Rodríguez R., Plaza-Calonge M.D.C., Anderson P., Rodríguez-Manzaneque J.C. (2018). ADAMTS1 Protease Is Required for a Balanced Immune Cell Repertoire and Tumour Inflammatory Response. Sci. Rep..

[66] Moayedfard Z., Sani F., Alizadeh A., Bagheri Lankarani K., Zarei M., Azarpira N. (2022). The Role of the Immune System in the Pathogenesis of NAFLD and Potential Therapeutic Impacts of Mesenchymal Stem Cell-Derived Extracellular Vesicles. Stem. Cell Res. Ther..

[67] Hansson G.K., Hermansson A. (2011). The Immune System in Atherosclerosis. Nat. Immunol..

[68] McLaren J.E., Calder C.J., McSharry B.P., Sexton K., Salter R.C., Singh N.N., Wilkinson G.W.G., Wang E.C.Y., Ramji D.P. (2010). The TNF-like Protein 1A-Death Receptor 3 Pathway Promotes Macrophage Foam Cell Formation in Vitro. J. Immunol..

[69] Jönsson-Rylander A.-C., Nilsson T., Fritsche-Danielson R., Hammarström A., Behrendt M., Andersson J.-O., Lindgren K., Andersson A.-K., Wallbrandt P., Rosengren B. (2005). Role of ADAMTS-1 in Atherosclerosis: Remodeling of Carotid Artery, Immunohistochemistry, and Proteolysis of Versican. Arterioscler. Thromb. Vasc. Biol..

[70] Lee C.W., Hwang I., Park C.-S., Lee H., Park D.-W., Kang S.-J., Lee S.-H., Kim Y.-H., Park S.-W., Park S.-J. (2011). Comparison of ADAMTS-1, -4 and -5 Expression in Culprit Plaques between Acute Myocardial Infarction and Stable Angina. J. Clin. Pathol..

[71] Gao Y., Wu W., Yu C., Zhong F., Li G., Kong W., Zheng J. (2016). A Disintegrin and Metalloproteinase with Thrombospondin Motif 1 (ADAMTS1) Expression Increases in Acute Aortic Dissection. Sci. China Life Sci..

[72] Laurent M.-A., Bonnier D., Théret N., Tufféry P., Moroy G. (2016). In Silico Characterization of the Interaction between LSKL Peptide, a LAP-TGF-Beta Derived Peptide, and ADAMTS1. Comput. Biol. Chem..

[73] Bourd-Boittin K., Bonnier D., Leyme A., Mari B., Tuffery P., Samson M., Ezan F., Baffet G., Theret N. (2011). Protease Profiling of Liver Fibrosis Reveals the ADAM Metallopeptidase with Thrombospondin Type 1 Motif, 1 as a Central Activator of Transforming Growth Factor Beta. Hepatology.

[74] Zhang H., Ma Y., Cheng X., Wu D., Huang X., Chen B., Ren Y., Jiang W., Tang X., Bai T. (2021). Targeting Epigenetically Maladapted Vascular Niche Alleviates Liver Fibrosis in Nonalcoholic Steatohepatitis. Sci. Transl. Med..

[75] Ambardekar A.V., Stratton M.S., Dobrinskikh E., Hunter K.S., Tatman P.D., Lemieux M.E., Cleveland J.C., Tuder R.M., Weiser-Evans M.C.M., Moulton K.S. (2021). Matrix-Degrading Enzyme Expression and Aortic Fibrosis during Continuous-Flow Left Ventricular Mechanical Support. J. Am. Coll. Cardiol..

[76] Prokesch A., Hackl H., Hakim-Weber R., Bornstein S.R., Trajanoski Z. (2009). Novel Insights into Adipogenesis from Omics Data. Curr. Med. Chem..

[77] Horodyska J., Reyer H., Wimmers K., Trakooljul N., Lawlor P.G., Hamill R.M. (2019). Transcriptome Analysis of Adipose Tissue from Pigs Divergent in Feed Efficiency Reveals Alteration in Gene Networks Related to Adipose Growth, Lipid Metabolism, Extracellular Matrix, and Immune Response. Mol. Genet. Genom..

[78] Rosen E.D., Hsu C.-H., Wang X., Sakai S., Freeman M.W., Gonzalez F.J., Spiegelman B.M. (2002). C/EBPalpha Induces Adipogenesis through PPARgamma: A Unified Pathway. Genes Dev..

[79] Watanabe M., Inukai K., Katagiri H., Awata T., Oka Y., Katayama S. (2003). Regulation of PPAR Gamma Transcriptional Activity in 3T3-L1 Adipocytes. Biochem. Biophys. Res. Commun..

[80] Tang Q.Q., Lane M.D. (2012). Adipogenesis: From Stem Cell to Adipocyte. Annu. Rev. Biochem..

[81] Gregoire F.M., Smas C.M., Sul H.S. (1998). Understanding Adipocyte Differentiation. Physiol. Rev..

[82] Hadrich F., Sayadi S. (2018). Apigetrin Inhibits Adipogenesis in 3T3-L1 Cells by Downregulating PPARγ and CEBP-α. Lipids Health Dis..

[83] Simão J.J., Cruz M.M., Abdala F.M., Bolsoni-Lopes A., Armelin-Correa L., Alonso-Vale M.I.C. (2022). Palmitoleic Acid Acts on Adipose-Derived Stromal Cells and Promotes Anti-Hypertrophic and Anti-Inflammatory Effects in Obese Mice. Pharmaceuticals.

[84] Zhou J., Li H., Xia X., Herrera A., Pollock N., Reebye V., Sodergren M.H., Dorman S., Littman B.H., Doogan D. (2019). Anti-Inflammatory Activity of MTL-CEBPA, a Small Activating RNA Drug, in LPS-Stimulated Monocytes and Humanized Mice. Mol. Ther..

[85] Herrero-Cervera A., Soehnlein O., Kenne E. (2022). Neutrophils in Chronic Inflammatory Diseases. Cell. Mol. Immunol..

[86] Josefs T., Barrett T.J., Brown E.J., Quezada A., Wu X., Voisin M., Amengual J., Fisher E.A. (2020). Neutrophil Extracellular Traps Promote Macrophage Inflammation and Impair Atherosclerosis Resolution in Diabetic Mice. JCI Insight.

[87] Van Avondt K., Maegdefessel L., Soehnlein O. (2019). Therapeutic Targeting of Neutrophil Extracellular Traps in Atherogenic Inflammation. Thromb. Haemost..

[88] Castanheira F.V.S., Kubes P. (2019). Neutrophils and NETs in Modulating Acute and Chronic Inflammation. Blood.

[89] Soehnlein O., Zernecke A., Eriksson E.E., Rothfuchs A.G., Pham C.T., Herwald H., Bidzhekov K., Rottenberg M.E., Weber C., Lindbom L. (2008). Neutrophil Secretion Products Pave the Way for Inflammatory Monocytes. Blood.

[90] Warnatsch A., Ioannou M., Wang Q., Papayannopoulos V. (2015). Inflammation. Neutrophil Extracellular Traps License Macrophages for Cytokine Production in Atherosclerosis. Science.

[91] Megens R.T.A., Vijayan S., Lievens D., Döring Y., van Zandvoort M.A.M.J., Grommes J., Weber C., Soehnlein O. (2012). Presence of Luminal Neutrophil Extracellular Traps in Atherosclerosis. Thromb. Haemost..

[92] Quillard T., Araújo H.A., Franck G., Shvartz E., Sukhova G., Libby P. (2015). TLR2 and Neutrophils Potentiate Endothelial Stress, Apoptosis and Detachment: Implications for Superficial Erosion. Eur. Heart J..

[93] Bristol J.A., Morrison T.E., Kenney S.C. (2009). CCAAT/Enhancer Binding Proteins Alpha and Beta Regulate the Tumor Necrosis Factor Receptor 1 Gene Promoter. Mol. Immunol..

[94] Crespo J., Cayón A., Fernández-Gil P., Hernández-Guerra M., Mayorga M., Domínguez-Díez A., Fernández-Escalante J.C., Pons-Romero F. (2001). Gene Expression of Tumor Necrosis Factor Alpha and TNF-Receptors, P55 and P75, in Nonalcoholic Steatohepatitis Patients. Hepatology.

[95] Divella R., Daniele A., DE Luca R., Mazzocca A., Ruggieri E., Savino E., Casamassima P., Simone M., Sabba C., Paradiso A. (2019). Synergism of Adipocytokine Profile and ADIPOQ/TNF-α Polymorphisms in NAFLD-Associated MetS Predict Colorectal Liver Metastases Outgrowth. Cancer Genom. Proteom..

[96] Wandrer F., Liebig S., Marhenke S., Vogel A., John K., Manns M.P., Teufel A., Itzel T., Longerich T., Maier O. (2020). TNF-Receptor-1 Inhibition Reduces Liver Steatosis, Hepatocellular Injury and Fibrosis in NAFLD Mice. Cell Death Dis..

[97] Yan W., Ding A., Kim H.-J., Zheng H., Wei F., Ma X. (2016). Progranulin Controls Sepsis via C/EBPα-Regulated Il10 Transcription and Ubiquitin Ligase/Proteasome-Mediated Protein Degradation. J. Immunol..

[98] Nam H.H., Jun D.W., Jang K., Saeed W.K., Lee J.S., Kang H.T., Chae Y.J. (2017). Granulocyte Colony Stimulating Factor Treatment in Non-Alcoholic Fatty Liver Disease: Beyond Marrow Cell Mobilization. Oncotarget.

[99] Liu M., Liu K., Chen D., Chen H., Sun K., Ju X., Lan J., Zhou Y., Wang W., Pang L. (2017). The Effect of Granulocyte Colony-Stimulating Factor on the Progression of Atherosclerosis in Animal Models: A Meta-Analysis. Biomed. Res. Int..

[100] Shannon P., Markiel A., Ozier O., Baliga N.S., Wang J.T., Ramage D., Amin N., Schwikowski B., Ideker T. (2003). Cytoscape: A Software Environment for Integrated Models of Biomolecular Interaction Networks. Genome Res..

[101] Doncheva N.T., Morris J.H., Holze H., Kirsch R., Nastou K.C., Cuesta-Astroz Y., Rattei T., Szklarczyk D., von Mering C., Jensen L.J. (2022). Cytoscape StringApp 2.0: Analysis and Visualization of Heterogeneous Biological Networks. J. Proteome Res..

[102] Szklarczyk D., Gable A.L., Lyon D., Junge A., Wyder S., Huerta-Cepas J., Simonovic M., Doncheva N.T., Morris J.H., Bork P. (2019). STRING V11: Protein–Protein Association Networks with Increased Coverage, Supporting Functional Discovery in Genome-Wide Experimental Datasets. Nucleic Acids Res..

[103] DAVID Functional Annotation Bioinformatics Microarray Analysis. https://david.ncifcrf.gov/.

[104] Tang Y., Li M., Wang J., Pan Y., Wu F.-X. (2015). CytoNCA: A Cytoscape Plugin for Centrality Analysis and Evaluation of Protein Interaction Networks. Biosystems.

[105] Bader G.D., Hogue C.W. (2003). An Automated Method for Finding Molecular Complexes in Large Protein Interaction Networks. BMC Bioinform..

[106] Piñero J., Ramírez-Anguita J.M., Saüch-Pitarch J., Ronzano F., Centeno E., Sanz F., Furlong L.I. (2020). The DisGeNET Knowledge Platform for Disease Genomics: 2019 Update. Nucleic Acids Res..

[107] Rappaport N., Twik M., Plaschkes I., Nudel R., Iny Stein T., Levitt J., Gershoni M., Morrey C.P., Safran M., Lancet D. (2017). MalaCards: An Amalgamated Human Disease Compendium with Diverse Clinical and Genetic Annotation and Structured Search. Nucleic Acids Res..

[108] Yu W., Clyne M., Khoury M.J., Gwinn M. (2010). Phenopedia and Genopedia: Disease-Centered and Gene-Centered Views of the Evolving Knowledge of Human Genetic Associations. Bioinformatics.

